# A novel approach to analyzing the evolution of SARS-CoV-2 based on visualization and clustering of large genetic data compactly represented in operative memory

**DOI:** 10.18699/vjgb-24-92

**Published:** 2024-12

**Authors:** A.Yu. Palyanov, N.V. Palyanova

**Affiliations:** A.P. Ershov Institute of Informatics Systems of the Siberian Branch of the Russian Academy of Sciences, Novosibirsk, Russia Research Institute of Virology, Federal Research Center of Fundamental and Translational Medicine, Novosibirsk, Russia Novosibirsk State University, Novosibirsk, Russia; Research Institute of Virology, Federal Research Center of Fundamental and Translational Medicine, Novosibirsk, Russia

**Keywords:** coronavirus, SARS-CoV-2, genome, variants, evolution, software system, big data, compact representation of data, analysis, visualization, коронавирус, SARS-CoV-2, геном, варианты, эволюция, программная система, большие данные, компактизация, анализ, визуализация

## Abstract

SARS-CoV-2 is a virus for which an outstanding number of genome variants were collected, sequenced and stored from sources all around the world. Raw data in FASTA format include 16.8 million genomes, each ≈29,900 nt (nucleotides), with a total size of ≈500 ∙ 109 nt, or 465 Gb. We suggest an approach to data representation and organization, with which all this can be stored losslessly in the operative memory (RAM) of a common PC. Moreover, just ≈330 Mb will be enough. Aligning all genomes versus the initial Wuhan-Hu-1 reference sequence allows each to be represented as a data structure containing lists of point mutations, deletions and insertions. Our implementation of such data representation resulted in a 1:1500 compression ratio (for comparison, compression of the same data with the popular WinRAR archiver gives only 1:62) and fast access to genomes (and their metadata) and comparisons between different genome variants. With this approach implemented as a C++ program, we performed an analysis of various properties of the set of SARS-CoV-2 genomes available in NCBI Genbank (within a period from 24.12.2019 to 24.06.2024). We calculated the distribution of the number of genomes with undetermined nucleotides, ‘N’s, vs the number of such nucleotides in them, the number of unique genomes and clusters of identical genomes, and the distribution of clusters by size (the number of identical genomes) and duration (the time interval between each cluster’s first and last genome). Finally, the evolution of distributions of the number of changes (editing distance between each genome and reference sequence) caused by substitutions, deletions and insertions was visualized as 3D surfaces, which clearly show the process of viral evolution over 4.5 years, with a time step = 1 week. It is in good correspondence with phylogenetic trees (usually based on 3–4 thousand of genome variant representatives), but is built over millions of genomes, shows more details and is independent of the type of lineage/clade classification.

## Abstract (ru)

Коронавирус SARS-CoV-2 – это вирус, для которого было собрано, секвенировано и сохранено рекордное количество вариантов генома из источников по всему миру. Нуклеотидные последовательности в формате FASTA включают 16.8 млн геномов, каждый длиной ≈29 900 нт (нуклеотидов), общим размером ≈500 ∙ 109 нт, или 466 Гб. Мы предлагаем способ представления данных, позволяющий разместить без потерь всю эту информацию в оперативной памяти (RAM) обычного персонального компьютера. Более того, будет достаточно всего ≈330 Мб. Выравнивание их всех относительно исходной референсной последовательности Wunah-Hu-1 позволяет представить каждый геном как структуру данных, содержащую списки точечных мутаций, делеций и вставок. Наша реализация такого представления данных привела к коэффициенту сжатия 1:1500 (для сравнения, упаковка данных с помощью популярного архиватора WinRAR дает степень сжатия только 1:62) и обеспечила возможность быстрого вычисления редакционного расстояния между различными вариантами генома. С помощью этого подхода, реализованного в виде программы на C++, мы провели анализ различных свойств набора геномов SARS-CoV-2, содержащихся в NCBI Genbank, собранных за 4.5 года (с 24.12.2019 по 24.06.2024). Были рассчитаны распределение числа геномов от числа неопределенных нуклеотидов “N” в них, число уникальных геномов и кластеров из идентичных геномов, а также распределение кластеров по размеру (числу идентичных геномов) и продолжительности (длине временного интервала между первым и последним геномом каждого кластера). Наконец, эволюция распределений числа изменений (редакционное расстояние между каждым геномом и референсной последовательностью), вызванных заменами, делециями и вставками, была визуализирована в виде 3D поверхностей, наглядно изображающих процесс вирусной эволюции в течение 4.5 лет, с интервалом в одну неделю. Такая визуализация хорошо соотносится с филогенетическими деревьями (обычно рассчитываемыми по 3–4 тыс. представителей вариантов генома), но строится на основе миллионов геномов, отображает больше деталей и не зависит от типа классификации линий/клад.

## Introduction

SARS-CoV-2 coronavirus, the very first sample of which, named Wuhan/Hu-1/2019, was collected on 24 December 2019 (Wu et al., 2020), caused the largest pandemic in the last 100 years (since the Spanish flu of 1918–1920). 4.5 years later, it is still persisting, evolving and being detected in people around the world, albeit in much smaller numbers than during the peak of the pandemic and with less severe consequences. However, usually infection rates rise again with the arrival of autumn, and 2024 is no exception. According to the World Health Association (https://data.who.int/dashboards/covid19/ cases, section “COVID-19 cases, country level trends”), by mid-September 2024 many countries have already started to experience an increase in the incidence of the disease. For example, in Russia, 26.7 thousand cases of SARS-CoV-2 infection were registered in July 2024, 24.7 thousand – in August, and already 62.2 thousand in the first half of September. In different countries there are certain features of the dynamics of the number of infections, depending on many factors, the analysis of interrelationships between which, in particular, we studied in (Palyanova et al., 2022, 2023).

SARS-CoV-2 virus samples obtained worldwide are sequenced and uploaded to databases, the largest of which are GISAID (gisaid.org) and NCBI Genbank (www.ncbi.nlm. nih.gov/sars-cov-2/) – as of 06.2024 they contain more than 16.7 ∙ 106 and more than 8.6 ∙ 106 SARS-CoV-2 genome samples, respectively. In comparison, human influenza virus, the earliest samples of which date back to 1905 in GISAID, has been represented by approximately 5.22 ∙ 105 genomes over more than a century. Given that the typical genome size of SARS-CoV-2 is 29.9 kb, the total volume of the genomes of this virus represented in GISAID is about 500 ∙ 109 nt (or 465 Gb), and in Genbank, about 258 ∙ 109 nt (241 Gb). All these data will not fit into the RAM of an average modern PC (16...64 Gb), while working with them directly from files located on a hard disc (HDD) or solid-state drive (SSD) will be much slower than from RAM. Read speeds from modern HDD/SDD/RAM have typical values of about 0.2, 3 and 50 Gb/s, respectively, so for significant data volumes and computational loads, working specifically with RAM is highly desirable.

Despite vaccination and drug treatment, there is currently no way to completely eliminate SARS-CoV-2 (Cui et al., 2023), so it is likely to remain with mankind for a long time, adding to the numerous list of more than 200 acute respiratory infections, including influenza, respiratory syncytial virus, rhinovirus, coronavirus, adenovirus, and other infections that cause catarrhal inflammation of the respiratory tract.

The longer a virus exists, the more changes accumulate in its genome – each new generation is obtained as a result of replication of viruses of the previous generation, in the process of which errors/changes may occur. Gene mutations can result in substitutions, deletions and insertions of one or more nucleotides, as well as in translocations, duplications and inversions of different parts of the gene. For example, point mutations occur spontaneously with frequencies of 10–8–10–6 for DNA viruses, and 10–6–10–4, for RNA viruses (Sanjuán, Domingo-Calap, 2016), the molecular machinery for replication of which (RNA polymerase) lacks an error-correcting mechanism (exonuclease). Coronaviruses and toroviruses, which do have it (Campagnola et al., 2022), are exceptions, as they have some of the largest genomes for RNA viruses, and too rapid accumulation of errors in them is apparently not desirable and does not favour virus survival.

According to (Amicone et al., 2022), the error rate during SARS-CoV-2 replication is 1.3 ∙ 10–6 ± 0.2 ∙ 10–6 substitutions per position per infectious cycle of cell infection (i. e. from virus entry into the cell to the exit of new virions from the cell). At the same time, the rate of evolutionary changes in the SARS-CoV-2 genome is estimated to be 8.9 ∙ 10–4 substitutions per position per year (Sonnleitner et al., 2022).

In addition to the above-mentioned mechanisms that can affect a single genome, there are also those that can create new combinations based on the genetic material of different genome variants. When two different variants of the same virus infect the same organism simultaneously (e. g., Delta and Omicron infection in the case of SARS-CoV-2 (Bolze et al., 2022)), they may interact during replication (Simon-Loriere Holmes, 2011), resulting in recombinants or reassortants (in the case of viruses with a segmented genome).

Regardless of which mechanism caused a particular change, the Levenshtein distance (also called edit distance) can be calculated for any pair of genomes of the considered virus, defined as the minimum number of single-character operations (substitutions, deletions, insertions) that need to be performed in the first genome to produce the second genome (or in the second genome to produce the first one – the result is the same). In other words, the Levenshtein distance sets a metric that defines the difference between two sequences of symbols. Thus, each variant of the SARS-CoV-2 genome out of the millions available can be compared to the original Wuhan-Hu-1 reference genome. For this purpose, it is necessary to perform a global alignment of all available sequences with respect to the reference, which we performed using the NextAlign/ NextClade software (https://github.com/nextstrain/nextclade) (Aksamentov et al., 2021). As a result, for each considered viral genome sequence, we calculated a list of changes (deletions, insertions, or point substitutions) that distinguish it from the reference genome sequence.

For a virus with a genome size of 30,000 nt, a single point substitution could occur at each of 30,000 positions and result in a change of an existing nucleotide (A, T, G, or C) to one of the three others, giving rise to 30,000 ∙ 3 = 90,000 different variants. A single insertion can be made at 30,001 positions – added either at the beginning or end of the sequence, or in any of the 29,999 spaces between the available nucleotides. It may contain any of the four letters of the alphabet, i. e. there are 120,004 different variations of such insertions. Finally, a deletion can occur in any of 30,000 positions, resulting in a number of variants equal to the number of positions. However, the deletions and insertions that leave the virus viable most often occur in blocks that are multiples of three, because otherwise such a change would result in a shift in the rea- ding frame, which in the vast majority of cases makes the genome non-viable. Thus, even one single change can be carried out in more than 240,000 different ways, although a significant part of them (especially those correspon- ding to deletions and insertions) will make the genome nonviable

The combination of two arbitrary point substitutions is already (240,000)2 = 5.8 ∙ 1010 and three – (240,000)3 = 1.4 ∙ 1016 variants, and this time among them there will be those with no reading frame shift (the result of changes – deletion or insertion of one triplet, i. e. three subsequent nucleotides). At the same time, the number of differences between some modern variants of SARS-CoV-2 and the reference genome already exceeds 200, and, for scale, the editorial distance between SARS-CoV-2 and the nearest genome of another virus – bat coronavirus, RaTG13 – is 1,136 (96.1 % of nucleotides match) (Zhou et al., 2020; Temmam et al., 2022). A number of questions about the space of variants of SARS-CoV-2 genetic sequences are discussed in more detail in (Palyanov, Palyanova, 2023), where, in particular, it is shown that thenumber of already realized variants of the virus is a negligible fraction of those that are potentially possible. Thus, both the continued monitoring of new SARS-CoV-2 variants and the analysis of the millions of genomes already accumulated over the past 4.5 years are of interest both from a practical point of view and for obtaining new fundamental knowledge in virology and epidemiology.

## Materials and methods

The results presented in this paper were obtained using a software package that we created to analyze the evolution of viruses. The C++ programming language and the development environment “Microsoft Visual Studio Community 2019” were used. One third-party software module required to perform global alignment of viral genomes was used – NextAlign by NextClade (https://github.com/nextstrain/nextclade/releases). Workstation based on Intel Core i7-10700K, 3.8 GHz, 8 cores, 32 Gb DDR4 operative memory was used for all computations.

The data for the analysis – the genetic sequences of SARS-CoV-2. The data used in this paper are the complete set of SARS-CoV-2 genomes contained in the Genbank database (www.ncbi.nlm.nih.gov/sars-cov-2/) on day 24.06.2024 (4.5 years since the collection of the first sample of this virus, Wuhan-Hu-1, 24.12.2019). The number of genomes is 8,641,740 and their total size is 242 Gb. The SARS-CoV-2 reference genome, which is 29,903 nt long, consists of a 5′ UTR (265 nt long), a CDS (which is 29,409 nt long and encodes 29 proteins (Bai et al., 2022)), and a 3′ UTR (229 nt long) (UTR is the untranslated region, CDS is the coding sequence). This dataset, which continues to grow over time, was analyzed to investigate evolutionary changes in SARS-CoV-2. Another source of data is the GISAID database, which includes a significant amount of the Genbank data; other genomes from it have yet to be analyzed and compared with the results for the Genbank genomes.

Data quality, preliminary data analysis and filtering. One of the first issues that arise when working with a set of nucleotide sequences of viral genomes is their quality. In particular, the sequences may contain not only letters encoding nucleotides (A, T, G, C), but also “N”s indicating unidentified, unknown nucleotides in the corresponding positions. The larger the number of “N”s, the greater the uncertainty, and the worse for the results of the analysis and their validity. In this regard, it is of course helpful to know how many such sequences there are in the dataset under study, and how many “N”s are present in them.

**Fig. 1.**
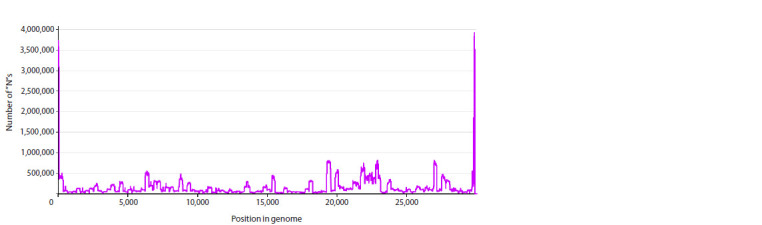
Frequency dependence of “N” occurrence rate as a function of position in the genome (abscissa axis), obtained by summarization over the full set of SARS-CoV-2 genetic sequences from Genbank in the interval from 24.12.2019 to 24.06.2024

Our calculations showed that out of the full sequence set (8,641,740), unidentified “N” nucleotides occur in 6,609,933 genomes (76.5 %) and are missing only in the remaining 2,031,807 (23.5 %). However, if we consider only CDS, the number of genomes without “N” almost doubles, reaching 3,742,117 (43.3 %). In addition, we plotted the dependence of the frequency of “N” occurrence on the position in the genome on the basis of the full set of sequences for which the global alignment was performed (Fig. 1).

As can be seen, there are two most significant peaks, at the beginning and at the end of the genome, corresponding to the non-coding regions of 5′ UTR and 3′ UTR, the total length of which is 1.65 % of the length of the whole genome. It is also known that in the genetic sequences of SARS-CoV-2 from GISAID and Genbank, the non-translational regions have a high variation in their lengths (Palyanov, Palyanova, 2023). Considering that the number of genomes in which “N” occurs in UTRs and does not occur in CDS is 22.5 % of all genomes, excluding UTR regions from consideration will almost double the set of data suitable for analysis (23.5 % of sequences in which “N” does not occur at all, neither in CDS nor in UTRs, will be supplemented with another 22.5 % in which “N” is present only in UTRs).

**Fig. 2.**
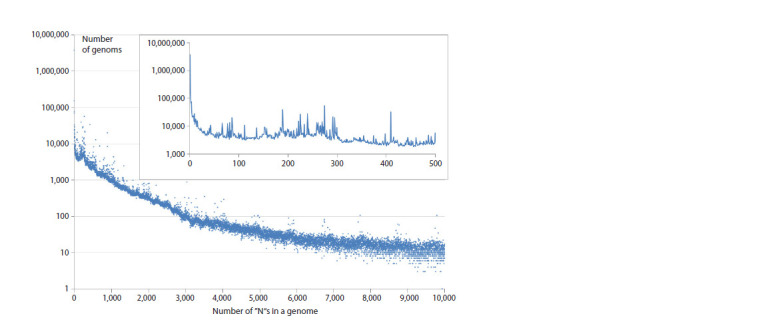
Distribution of genomes by the number of unidentified “N” nucleotides in them. Calculated over the full set of SARS-CoV-2 genetic sequences from Genbank between 24.12.2019 and 24.06.2024. The inset shows the same relationship, but with higher resolution, for the number of “N”s in the genome between 0 and 500.

Depending on what is the distribution of genomes by the number of “N”s contained in their CDS, we can either use those genomes with only a few “N”s (in comparison with editing distance values of the order of 100 point substitutions, this is an insignificant value, although their presence introduces some uncertainty), or use only those genomes with no “N”s in the CDS. Having plotted the mentioned distribution (Fig. 2), we found that exactly one “N” is present in 1.8 % of genomes, two and three – in 0.8 and 0.9 %, respectively, and the number of “N”s between 1 and 10 per genome – in 5.4 %. As a result, at this stage it was decided to work only with genomes in which “N” is absent in CDS, and to use only CDS in calculations, excluding 5′ UTRs and 3′ UTRs.

Methods, algorithms and data structures. To construct global alignments of all genomes (using the Wuhan-Hu-1 genome as a reference), we used the console version of NextAlign (running in multithreaded mode), called with the necessary parameters being passed from our software system. This happens during the first run or when the alignments need to be recalculated (e. g., if a different genome dataset is used). On the full dataset mentioned above, consisting of 8.6 million SARS-CoV-2 genomes, the calculation of alignments takes about one day on a workstation with an Intel Core i7- 10700K @ 3.8 GHz processor (8 cores, 16 threads) and 32 Gb of RAM (DDR4, 3,600 GHz). The output of NextAling for all calculated alignments is stored in files on the hard drive in the working directory of the program, which are then used by our system as input data used for analysis. The files are composed as tables with several dozen columns, including various genome characteristics and metadata, as well as lists of mutations, deletions and insertions that distinguish the considered genome from the reference genome.

As the data are read, a list of structures is dynamically generated in the computer’s RAM, each of which includes the virus name, collection date, geographical data, and a complete set of changes that distinguish the current variant from the reference genome:

• a list of point mutations (single position substitutions), each element of which contains the position number in the genome corresponding to the mutation and a letter encoding the nucleotide that appears at that position as a result of the substitution (the previous nucleotide that was present before the mutation is not stored – it can always be read from the corresponding position in the reference genome if necessary);

• a list of deletions, each of which is defined by two numbers – the positions of the beginning and the end of the deletion;

• a list of insertions, each of which is defined by the position in the genome immediately after which the insertion took place, as well as by the inserted sequence

This organization of the data allows two arbitrary genomes to be compared quickly and easily. It is especially quick to determine whether they are identical or not. Instead of comparing each of the 29,409 positions of the first and second genomes, it is enough to simply compare the number of elements in their lists of point mutations, deletions and insertions – at least one difference makes it clear that the genomes are different. However, in this way it is possible to obtain not only the result of genome comparison, but also to calculate the editorial distance between them. Matching elements of the difference lists do not contribute to the difference between the genomes, whereas each element of difference from the reference, present in one genome and absent in the other, adds a corresponding amount of difference. Each substitution that occurred at the same position in both genomes but resulted in substitutions to different nucleotides also, of course, adds +1 to the edit distance. Given that the list sizes are quite small, the comparison is much faster than comparing two genomes without prior alignment.

Our proposed method of compact representation of nucleotide sequences of related genomes in computer memory has much in common with the compression method that represents sequences in the form of a phylogenetic tree with substitutions on edges. Moreover, the very representation of each genome as a set of changes that need to be made to go from the reference to the genome under consideration is based on the same data representing the structure of the phylogenetic tree built on the basis of multiple alignments of the sequences under consideration.

The peculiarity of our implementation is that the data structure in a PC operative memory, representing the set of sequences under consideration, is not a phylogenetic tree, but instead is a list of its “leaves” sorted in chronological order, by the date of obtaining samples. For such tasks as analyzing not just the available spectrum of virus variants, but its evolutionary changes taking into account the time of their emergence, our approach provides a significant advantage in the speed of data access. The point is that it allows us to move along the time axis simply by increasing or decreasing the index of an array element consisting of time-ordered genomes. And in the tree representation, the search for all genomes corresponding to a certain year, month and day, in general case, may require traversing the whole tree, and so for each genome variant being processed. At the same time, each “leaf” in our approach contains all the information about its “branch” of the tree, which allows one to easily and quickly calculate the editing distance for any pair of genome variants.

## Results

Cluster structure of the genomes dataset

In the course of the study, we noticed that among the genomes under consideration, there are quite often genomes with CDSs that are 100 % identical to each other, while the date of sample collection, geographical data, and other metadata most often are different. By adding a function to our software system to identify all genomes with identical CDSs (and combine them into “clusters”), we have divided the entire set of genomes into such groups. The statistics on them turned out to be as follows from the Table.

**Fig. 3.**
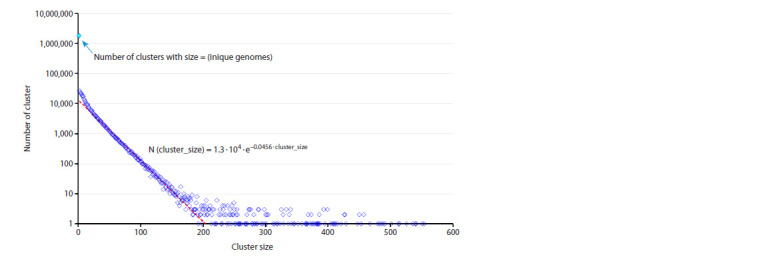
The dependence between the number of clusters and their size for the set of SARS-CoV-2 genomes from Genbank in the interval from 24.12.2019 to 24.06.2024. In the interval of cluster size values from 20 to 200, it is well approximated by an exponent with the parameters indicated in the Figure.

**Fig. 4.**
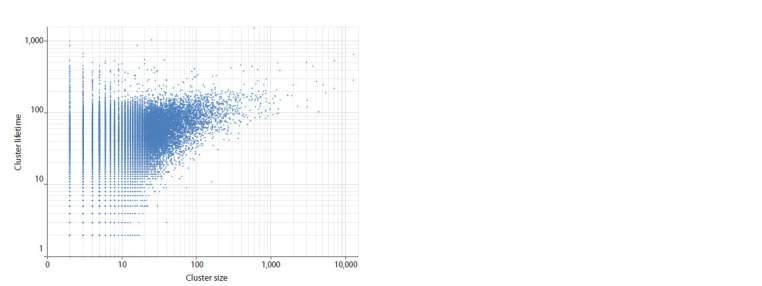
Dot cloud representing the set of SARS-CoV-2 genomes from Genbank (between 24.12.2019 and 24.06.2024) using their “cluster size” and “cluster lifetime” features.

Also, we calculated the relationship between cluster size and the number of clusters of a particular size (Fig. 3). At the same time, there is no obvious dependence between cluster sizes and their lifetime; the distribution is a cloud of points, most of which is concentrated in the region from 1 to 1,000 on the “cluster size” axis and from 1 to 500 on the “cluster lifetime” axis (Fig. 4).

**Fig. 5.**
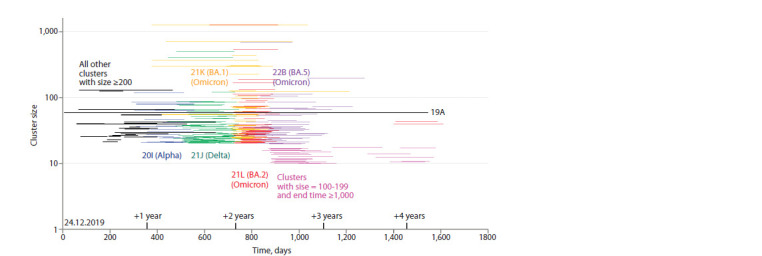
The largest clusters (size ≥200) and their existence intervals (lines connecting the day of the first appearance of the genome variant representing this cluster and the day when the last sample with the same genome was taken). All clusters of size ≥200 depicted in the same colour belong to the same genetic lineage, the name of which is also displayed with that colour. All genomes belonging to the same line are equal, and genomes of different lines differ between each other. The exceptions are “all other clusters with size ≥200” depicted in black, which represent a collection of different genetic lineages (19A, 20A, 20B, 20C, 20E and 20F), and clusters of size 100–199 depicted in magenta (which have an end-of-life date ≥1,000 days from the date of collection of the first SARS-CoV-2 genome, 24.12.2019).

We also plotted (Fig. 5) all clusters of size ≥ 200 on the time axis and represented them as lines, with the beginning and end corresponding to the clusters’ existence intervals. In addition, we added all clusters of size 100–199, the end of the existence interval of which has a value ≥ 1,000 days since the first SARSCoV- 2 genome was obtained. This set of clusters covers the entire timeline, although there are clearly other clusters in the interval between 3 and 3.5 years, but all of them are smaller than those shown in the Figure 5. The individual line “19A”, the longest in the Figure 5, corresponds to the cluster with the longest lifetime (1,539 days or 4.2 years) mentioned in Table. In this regard, the genetic line 19A, which has survived for such a long time, appears to be quite interesting. This genome variant was detected quite stably both at the beginning of the pandemic and in 2023–2024.

A novel approach to visualizing evolutionary changes in SARS-CoV-2

**Fig. 6.**
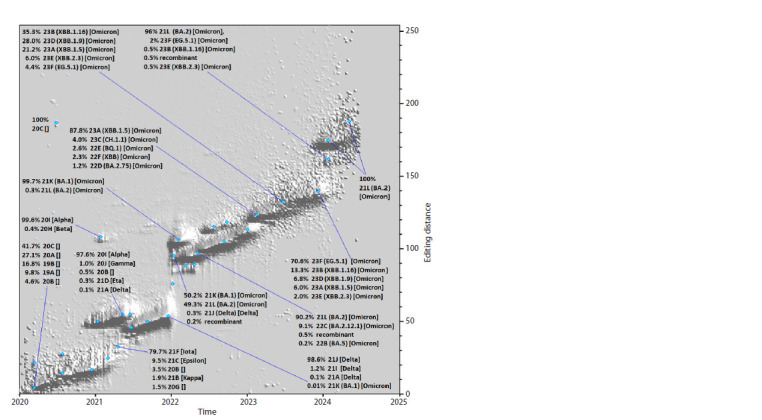
The landscape of the space of SARS-CoV-2 variants “visited” by the virus variants during the period from 24.12.2019 to 24.06.2024, projected on three axes: OX – time (sampled at 1 week), OY – difference (editing distance) between a point on the landscape and a reference genome, OZ – fraction of genomes corresponding to a point with certain X and Y values, referred to the total number of genomes collected in week X. Such normalization is necessary due to the fact that the dependence of the number of genome samples collected in one or another week all over the world has significant changes over time, and without the proposed normalization the weekly distribution in case of, for example, 100 genomes will be completely invisible in comparison with some other week represented by 10,000 genomes, whereas even for 100 genomes distributions are quite informative.

Having obtained the ability to quickly calculate the editorial distance between a pair of any nucleotide sequence variants, we did it for the whole set of SARS-CoV-2 genomes from Genbank for 4.5 years. Thus, for each genome, there is a pair of numbers – the collection date of the genome sample and the editing distance between it and the reference. Sometimes different variants of genomes appear to possess the same pair of values “date + editorial distance”, because the same value of the editing distance can be a result of, for example, a deletion of length 30, an insertion of the same length, or 30 point mutations scattered throughout the genome. If we introduce a third value – the number of cases in which the genome variant has a certain editing distance between it and the reference and a certain date of sample collection – then we can calculate triples of these values on the basis of the complete set of SARS-CoV-2 genomes and display them as a surface, which we did (Fig. 6).

In Figure 6, we have marked a number of interesting landscape elements with blue dots, for each of which the corresponding spectrum of variants has been calculated. For many of them, it was possible to place this information in the figure. The landscape shows regions with different features – narrow extended “mountain ranges” with a beginning, an end and a characteristic slope angle (which has close values for most of them), apparently related to the rate of accumulation of changes in the genome appearing due to point nucleotide substitutions.

**Fig. 7.**
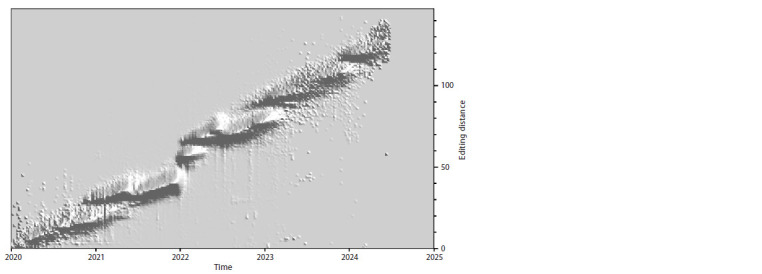
The landscape of evolutionary changes based on contributions from point substitutions only.

**Fig. 8.**
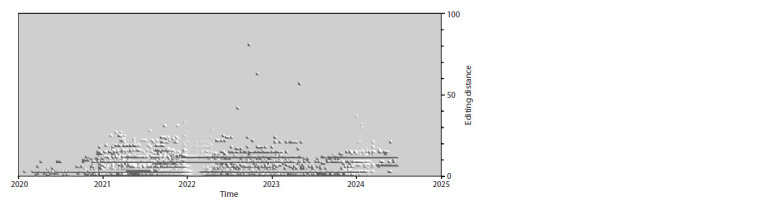
The landscape of evolutionary changes based on contributions from point insertions only.

**Fig. 9.**
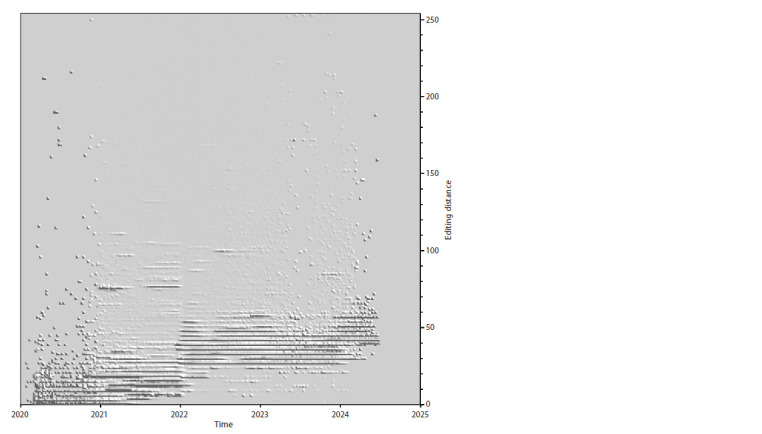
The landscape of evolutionary changes based on contributions from point deletions only.

There are also regions in which the editing distance for the entire set of variants existing at a given point in time changes rapidly and significantly in the mean value or experiences branching, splitting into several parallel, visually distinguis Statistical hable paths. Assuming that such abrupt and significant changes could be due to deletions, insertions, or recombination events, we constructed three more figures similar to Figure 6, for which we used not the full value of the editing distance, but its three separate contributions – from a set of point substitutions (Fig. 7), deletions (Fig. 8), and insertions (Fig. 9). Of the total number of genomes (with no “N” in CDS), 3,742,117, the number of genomes with mutations relative to the reference was 3,741,518, the number of genomes with deletions was 3,520,077, and the number of genomes with insertions was 528,414.

As can be seen in Figures 7–9, the dynamics of evolutionary changes introduced into SARS-CoV-2 genomes by different evolutionary mechanisms differ quite significantly for substitutions, deletions, and insertions. From Figure 7 we can conclude that the accumulation of the number of point mutations (substitutions) increases linearly on a large time scale (especially in the case of following the upper boundary of the evolutionary pathway region). In 4.5 years, about 130 point mutations were accumulated, i. e., the growth rate was about 29 nt per year (≈2.4 per month or ≈0.08 per day). The impact of deletions is also significant, and their number also grows near linearly with time, but at a slower rate of about 50 nt in 4.5 years, i. e., about 11 per year or slightly less than one per month. And finally, insertions, as can be seen from Figure 8, make a noticeably smaller contribution than substitutions and deletions, which, with the exception of the first year, practically does not grow with time – it stays at the level of +20 nt relative to the reference genome (although the content of these insertions, in principle, can change over time, from year to year).

## Discussion

We performed a number of evaluations and calculations, mainly using software tools developed by us, to improve our understanding of which features and trends related to the evolution of SARS-CoV-2 coronavirus genetic sequences can be found and effectively used. The proposed method of visualizing landscapes of evolutionary changes allowed us to display many details and features that are not visible, for example, on a phylogenetic tree. At the same time, rapid changes in the evolutionary trajectory accompanied by stepwise changes in the value of the editing distance, as can be seen from Figure 6, are usually accompanied by a change of the dominant virus variant in the population. Thus, for example, for one of such “jumps” on the evolutionary landscape, we were able to see the genetic lineages “Iota”, “Epsilon”, and “Kappa” in the corresponding spectrum of variants (approximately in the first quarter of 2022).

The observed non-growing contribution to the editing distance of inserts, previously mentioned for the results related to Figure 8, may be due to the fact that too many inserts may compromise the stability of the virus. Increasing the number of inserts increases the physical size of the genome and thus may impair its ability to fit inside the protein envelope, which is presumably designed to contain an object of a certain size. Thus, in the course of evolution, the number of insertions relative to the reference genome may increase, but not exceeding 20–30 nt. If some of the new variants turn out to be more adapted than their predecessors, they may soon displace them. It can be seen that only at the beginning of 2024, variants with no insertions at all are disappearing – apparently, due to the fact that during 4 years of evolution, such insertions were found, which turned out to be noticeably more adaptable than variants with no insertions at all and became established in the population. It can also be seen in Figures 8 and 9 that the values of contributions to the editing distance from deletions and insertions in most cases have a length multiple of three, which has an obvious explanation – other length variants will lead to a shift of the reading frame during the synthesis of proteins encoded in the genome and, in most cases, to non-viable copies of genomes, the virions of which cannot be assembled.

## Conclusion

As a result of this work we have obtained the following main results:

• a method of representing nucleotide sequences of virus genomes, which provides their extremely compact representation in computer memory, has been proposed and implemented as a computer program. On the example of SARS-CoV-2 coronavirus it is has been shown that compression of ≈1,500 times is provided. Using it for transmission of genetic data over the internet could reduce the load on servers and network traffic by a corresponding number of times (especially when transmitting large datasets);

• for the complete set of SARS-CoV-2 genomes (without “N”s in CDS), the presence of clusters of completely identical genomes has been investigated. It has been found that their size can exceed 10,000, and their lifetime can cover up to several hundred days;

• a new way of displaying the evolutionary dynamics of viruses in the form of a landscape visualizing the projection of the space of virus genome variants on three axes – time (T), editing distance to the reference genome (D), and the fraction of genomes (P) at each point (T, D) in the total number of genomes corresponding to a given T is proposed;

• it is also shown that the landscape constructed for D (calculated as the sum of contributions from point mutations, deletions and insertions) can be divided into three separate landscapes calculated separately for each of the contributions. Each of them has a different character, allowing the contribution and impact of each of the mentioned mechanisms on virus evolution to be estimated. The constants characterizing each of the mechanisms and the rate of changes acquired due to it have been calculated;

• the fact that the lineage 19A has existed for the longest time compared to the other clusters, covering the entire pandemic period, allows us to propose to create new vaccines against SARS-CoV-2 on the basis of this lineage, as it retains the greatest competitiveness compared to the other variants, and thus contains the most characteristic features of this virus that can be recognized by the immune system.

Our further plans include investigation of the possibilities of the proposed method of evolution visualization in more detail, but we can already state that it seems to be useful, has the potential for further use and development, and can be applied not only to SARS-CoV-2, but also to other viruses. The same can be said about the proposed method of compact representation of viral genomes, the application of which in all areas related to the storage, network transmission, processing and analysis of a large number of variants of related genomes (both viruses and living organisms) will provide significant advantages.

## Conflict of interest

The authors declare no conflict of interest.
